# A CRISPR/Cas9 mediated point mutation in the alpha 6 subunit of the nicotinic acetylcholine receptor confers resistance to spinosad in *Drosophila melanogaster*

**DOI:** 10.1016/j.ibmb.2016.04.007

**Published:** 2016-06

**Authors:** Christoph T. Zimmer, William T. Garrood, A. Mirel Puinean, Manuela Eckel-Zimmer, Martin S. Williamson, T.G. Emyr Davies, Chris Bass

**Affiliations:** a Rothamsted Research, Biological Chemistry and Crop Protection Department, Harpenden, UK; b College of Life and Environmental Sciences, Biosciences, University of Exeter, Penryn Campus, Penryn, Cornwall TR10 9FE, UK

**Keywords:** CRISPR, Nicotinic acetylcholine receptor, nAChR, Spinosad, Insecticide resistance

## Abstract

Spinosad, a widely used and economically important insecticide, targets the nicotinic acetylcholine receptor (nAChRs) of the insect nervous system. Several studies have associated loss of function mutations in the insect nAChR α6 subunit with resistance to spinosad, and in the process identified this particular subunit as the specific target site. More recently a single non-synonymous point mutation, that does not result in loss of function, was identified in spinosad resistant strains of three insect species that results in an amino acid substitution (G275E) of the nAChR α6 subunit. The causal role of this mutation has been called into question as, to date, functional evidence proving its involvement in resistance has been limited to the study of vertebrate receptors. Here we use the CRISPR/Cas9 gene editing platform to introduce the G275E mutation into the nAChR α6 subunit of *Drosophila melanogaster*. Reverse transcriptase-PCR and sequencing confirmed the presence of the mutation in *Dα6* transcripts of mutant flies and verified that it does not disrupt the normal splicing of the two exons in close vicinity to the mutation site. A marked decrease in sensitivity to spinosad (66-fold) was observed in flies with the mutation compared to flies of the same genetic background minus the mutation, clearly demonstrating the functional role of this amino acid substitution in resistance to spinosad. Although the resistance levels observed are 4.7-fold lower than exhibited by a fly strain with a null mutation of *Dα6,* they are nevertheless predicated to be sufficient to result in resistance to spinosad at recommended field rates. Reciprocal crossings with susceptible fly strains followed by spinosad bioassays revealed G275E is inherited as an incompletely recessive trait, thus resembling the mode of inheritance described for this mutation in the western flower thrips, *Frankliniella occidentalis*. This study both resolves a debate on the functional significance of a target-site mutation and provides an example of how recent advances in genome editing can be harnessed to study insecticide resistance.

## Highlights

• The nAChR alpha6 subunit of *Drosophila melanogaster* has been successfully mutated *in vivo* by CRISPR/Cas9 genome editing.
• We demonstrate that the G275E mutation in *Dα6* confers significant resistance to spinosad.
• G275E in *Dα6* resembles the phenotype reported for this particular mutation in *Frankliniella occidentalis*.

## Graphical abstract

**Figure:**
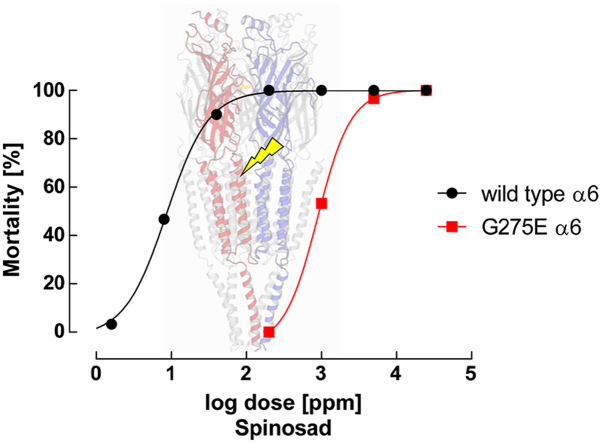

## 1 Introduction

Insecticide resistance is an exceptional example of rapid adaptive evolution and has provided a range of insights into the diversity of genetic alterations that occur in response to novel selective pressures. A common mechanism of insect resistance to insecticides, termed ‘target-site resistance’ involves alterations (mutations) in the insecticide target protein that reduce its sensitivity to insecticide. Target-site resistance most frequently involves point mutations at select positions in the target receptor as small changes to proteins are least likely to disrupt their, usually important, native function (ffrench-Constant et al., 1998).

The nicotinic acetylcholine receptor (nAChR) α6 subunit is a rare example of an insecticide target-site that can tolerate more radical alterations as it appears to be a redundant target (Perry et al., 2007). Insect α6-containing receptors are the target of spinosad, a macrocyclic lactone bio-insecticide derived from secondary metabolites of the soil bacteria *Saccharopolyspora spinosa*. Several lines of research indicate that spinosad binds at a site distinct from the neonicotinoid insecticides one exerting its effect through an allosteric mechanism (Orr et al., 2009, Puinean et al., 2013, Salgado and Saar, 2004).

The first resistance-conferring mutation described in the gene encoding this subunit was a null mutation of *Dα6*, in the fruit fly, *Drosophila melanogaster*, which was found to result in >1000-fold resistance to spinosad (Perry et al., 2007). Significantly, mutant flies were viable and displayed no obvious fitness deficit. Based on this finding Perry et al. predicted that loss of function mutations in *Dα6* orthologues may lead to spinosad resistance in field populations of insect pests (Perry et al., 2007). This prediction has held true with a range of genetic alterations in nAChR *α6* now described in several insect crop pests that result in truncated non-functional proteins. For example, several mutations resulting in mis-splicing and premature stop codons in nAChR α6 transcripts are associated with spinosad resistance in the diamondback moth, *Plutella xylostella*, and the oriental fruit fly *Bactrocera dorsalis* (Baxter et al., 2010, Hsu et al., 2012, Rinkevich et al., 2010).

Recently, however, spinosad resistance in several insect pest species has been associated with the same non-synonymous point mutation in exon 9 of the α6 nAChR that does not result in loss of function (Bao et al., 2014, Puinean et al., 2013, Silva et al., 2016). This mutation was initially described in western flower thrips, *Frankliniella occidentalis* (Puinean et al., 2013), and results in the replacement of a glycine (G) residue at position 275 observed in susceptible strains with a glutamic acid (E) in resistant strains. The same substitution was subsequently described in spinosad resistant melon thrips, *Thrips palmi* (Bao et al., 2014), and, very recently, tomato leafminer, *Tuta absoluta* (Silva et al., 2016).

The causal role of this mutation in resistance was recently questioned by Hou et al., after these authors characterized the nAChR α6 from three susceptible and two spinosad resistant strains of *F. occidentalis* from China and the USA and observed no difference in the cDNA sequences of resistant and susceptible thrips (Hou et al., 2014).

Functional validation of mutations in insect nAChRs, such as G275E, has been hampered by difficulties encountered in their expression in heterologous systems. Indeed, as a surrogate, Puinean et al. examined the potential effects of the G275E using human nAChR α7, a model receptor that readily forms functional homomeric receptors when expressed in heterologous systems (Puinean et al., 2013). Expression of the analogous mutation (A275E) in human α7 in *Xenopus oocytes* was found to abolish the modulatory effects of spinosad but had no significant effect upon activation by the natural ligand acetylcholine (Puinean et al., 2013). Although this evidence supports a causal role for the mutation, no functional validation of G275E in an insect system has been performed to date.

The type II clustered regular interspersed short palindromic repeat (CRISPR)/associated protein-9 nuclease (Cas9) system has recently emerged as an efficient tool to introduce precise, targeted changes to the genome of living cells. The CRISPR/Cas9 system exploits the RNA-guided endonuclease function of Cas9 to introduce double-stand breaks (DSBs) at defined loci that are then repaired by either nonhomologous end joining (NHEJ) or homology-directed repair (HDR). To introduce single nucleotide replacements in target genes HDR is exploited to repair DSBs by providing homologous sequence from a donor template such as a ssOligo or plasmid. CRISPR/Cas9-mediated editing of the genome of *D. melanogaster* has now been reported (Port et al., 2014) and the first use of this technology to introduce a resistant mutation into a controlled genetic background has also recently been described (Somers et al., 2015).

Here we describe the use of the CRISPR/Cas9 system to introduce the G275E mutation into *D. melanogaster* and demonstrate the causal role of this amino acid replacement in resistance to spinosad.

## 2 Material and methods

### 2.1 *D. melanogaster* strains

Fly strains described in this study were maintained on standard food (Bloomington formulation) at 24 °C. Fly strains deficient for DNA ligase 4 (#28877, genotype w^1118^ Lig4^169^), expressing endonuclease Cas9 (#51324, genotype w^1118^; PBac{y[+mDint2] = vas-Cas9}VK00027), deficient for the alpha6 subunit of the nAChR (#556, genotype w*; Df(2L)s1402, P{w[+mC] = lacW}s1402/CyO) and throughout the manuscript referred to as ‘Dα6 KO’, as well as the wildtype strain Canton-S (#1, wildtype) were sourced from the Bloomington Drosophila Stock Center at Indiana University, USA. The lig4 deficient strain and the Cas9 expressing strain were crossed and consecutive PCR assisted sibling mating allowed the rescue of a strain homozygous for both traits (genotype *w*^1118^ *Lig4*^169^; Bac{y[+mDint2] = vas-Cas9}VK00027), hereafter called ‘lig4 KO Cas9’.

### 2.2 gRNA design, template oligo and plasmid construction

**Fig. 1:**
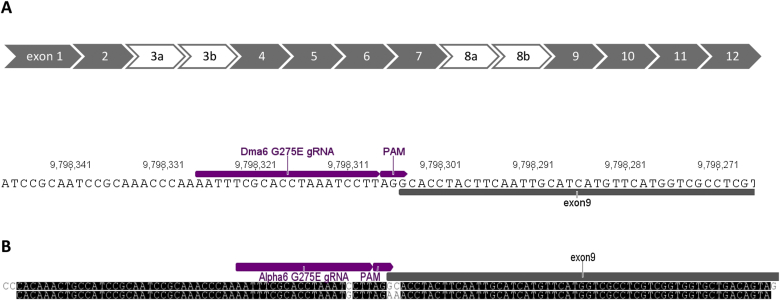
Dα6 exon organisation, gRNA target site and HDR template. A) Exon organisation, white arrows indicating alternative exon 3a/b and 8a/b (top) and 20 nt CRISPR/Cas9 target site as indicated by the large arrow above the sequence followed by the ‘ngg’ PAM motif (bottom). B) Nucleotide alignment of the 110 nt HDR template with Dα6. The single C/G substitution prevents re-cleavage of Cas9 through a mismatch in the gRNA seed sequence (last 12 nt of gRNA) and the double substitution GC/AA introduces a codon substitution from glycine (G) to glutamatic acid (E).

The gRNA was designed using the online platform http://www.flyrnai.org/crispr2/ (Housden et al., 2015). A region spanning ∼250 bp either side (>2L:9798031-978511) of the position of the desired point mutation was specified for the design. Based on the number of predicted off-targets a gRNA (>2L 2L:9798305.9798324 (- strand) AATTTCGCACCTAAATCCTT) was chosen as this was the only gRNA predicted to have no off-targets in combination with the predicted cutting site in close proximity to the nt position of the desired mutation (2L:9798305/9798306) (Fig. 1A). A gRNA expression plasmid was generated by cloning annealed gRNA oligonucleotides (Table 1) into the pCFD3: U6:3-gRNA plasmid (addgene #49410) as described elsewhere (Port et al., 2014). A single stranded oligonucleotide of 110 nt in size (template ssOligo) was designed to serve as a template for HDR following the Cas9 induced double strand break. The template ssOligo was designed with a dinucleotide polymorphism (Fig. 1B) which when incorporated into the genome would result in an alternate codon encoding glutamatic acid (E) instead of the native glycine (G) at AA position 275 of Dα6 (accession number NT_033779, AA count differs by 26 AA as position 275 refers to the protein after cleavage of the signal peptide). The template also contained a single nucleotide polymorphism (SNP) corresponding to intronic sequence just upstream of the above mutation site to prevent re-cleavage from Cas9 after incorporation (Fig. 1B).

### 2.3 Embryo injections and rescue of CRISPR mediated mutations

Embryos were collected from lig4 KO Cas9 flies and injections were carried out on an inverted microscope (eclipse Ti—U Nikon, Japan) equipped with a 10×/0.25 lens, 10×/22 eyepiece and fluorescence illumination. The injection mix comprised 0.5× phosphate buffer (pH 6.8, 0.05 mM sodium phosphate, 2.5 mM KCL) containing 200 ng μl^−1^ gRNA expression plasmid, 1 μg μl^−1^ template ssOligo and 200 mg L^−1^ fluorescein sodium salt to improve the monitoring of injections. The mix was delivered by a micromanipulation set-up consisting of a motorised micromanipulator TransferMan NK2 (Eppendorf, Hamburg, Germany) and a Femto Jet express microinjector (Eppendorf, Hamburg, Germany). Injection needles were prepared according to Miller et al. (2002) and injections into non-dechorionated embryos was carried out according to Miller et al. (2002) with modifications described previously (Arnoult et al., 2013). Emerging flies were crossed to flies of the Dα6 deficient strain, Dα6 KO with the curly wing phenotype (Cy), as we expected both knock-outs as well as the point mutation to be a recessive trait based on previous reports regarding spinosad resistance conferred by this gene (Perry et al., 2007, Puinean et al., 2013). Crossings were performed under spinosad selection pressure subjecting the flies to standard media to which spinosad was added to reach a final concentration of 250 μl L^−1^ active ingredient. This concentration does not affect adult flies but is sufficient to prevent the development of susceptible flies in the next generation (see Supplementary Fig. 1). Developing flies were screened for Cy and Cy males were crossed to virgin Cy Dα6 KO females. 5 days after the crossings were set up the males were retrieved for PCR and sequencing and the females were discarded. The emerging flies were again screened for curly wings and sibling mating was set up to select against Cy in the next generation.

### 2.4 PCR analysis and sequencing

DNA was extracted from single adult flies using 20 μl microlysis plus extraction buffer (Microzone Ltd., Haywards Heath, Sussex, UK) following the manufacturer's recommended protocol for tough cells. A typical PCR (20 μl) contained 0.5 μM of each primer (Table 1), 2 μl extracted DNA, 10 μl DreamTaq (Thermo Fisher, Waltham, MA, USA) containing Taq polymerase, 2× PCR buffer and 4 mM MgCl_2_ (2 mM final concentration). Cycling conditions were 95 °C for 2 min followed by 35 cycles of 95 °C for 20sec, 57 °C for 20sec and 72 °C for 1 min, and a final elongation at 72 °C for 5 min. To verify the mutation at the level of the transcript total RNA was extracted from pools of 5 adults using the Isolate II RNA mini kit (Bioline, London, UK) following the manufacturer's protocol. cDNA was transcribed using a cDNA synthesis kit (PCRBIO, London, UK) and 1 μl was subsequently used in a PCR reaction using cycling conditions and master mix as described above but using a nested approach. For the primary PCR primer ‘a6 cDNA F1’ (Table 1) was used in combination with ‘a6 gDNA/cDNA R’ with 20 cycles of thermocycling and 0.5 μl of the primary reaction was subsequently used as a template in the secondary PCR with the primers ‘a6 cDNA F1’ and ‘a6 gDNA/cDNA R’ with 15 cycles of thermocycling. PCR products were verified by agarose gel electrophoresis prior to PCR cleanup and sequencing which was carried out by Eurofins Genomics (Ebersberg, Germany). Sequence analysis and protein alignments were done with Geneious R8 (Biomatters, Auckland, New Zealand).

### 2.5 Insecticide bioassays and statistical analysis

3–5 day old adult females were used in insecticide bioassays to assess the susceptibility of different fly strains and crossings to the commercial formulation of spinosad (Conserve^®^, 11.6% spinosad in SC formulation, Dow Agrosciences, Indianapolis, IN, USA). The flies were subjected to the insecticide in a contact/feeding bioassay. Standard Drosophila vials (#789001, Dutscher Scientific, Brentwood, Essex, UK) were filled with agar solution (4 ml/vial) containing 2% w/v agar (Dutscher Scientific, #789150), 1.2% w/v food grade sucrose and 0.4% v/v glacial acetic acid (Sigma Aldrich, St. Louis, MO, USA). A 5000 mg L^−1^ spinosad stock solution was prepared by adding 43.1 μl Conserve^®^ per ml^−1^ tap water and a 5-fold serial dilution was prepared to achieve a concentration range from 5000 mg L^−1^ to 0.32 mg L^−1^ spinosad in tap water. 18 h prior to bioassay the agar vials were spread with 100 μl of spinosad solution and vortexed vigorously. For each concentration vials were prepared in triplicate for each fly strain. Flies were anaesthesised with CO_2_ and 10 female flies added to each vial. The vials were kept upside down until all flies became active to avoid flies getting trapped in agar. The bioassay was assessed after 48 h, dead flies as well as seriously affected flies i.e. those displaying no coordinated movement, that were unable to walk up the vial, or unable to get to their feet were cumulatively scored as ‘affected’. The raw data was corrected for control mortality using Abbott's formula (Abbott, 1925) and lethal concentrations LC_50_ and LC_95_ were calculated by probit analysis using the Polo Plus software v.1 (LeOra Software, Berkeley, CA, USA). The mode of inheritance was calculated according to Stone applying the respective LC values (Stone, 1968). Non-linear log dose-response curves were generated in Graphpad Prism 6.07 (Graphpad Software Inc., La Jolla, CA, USA).

## 3 Results

### 3.1 CRISPR mediated G275E replacement in *D. melanogaster*

**Fig. 2:**
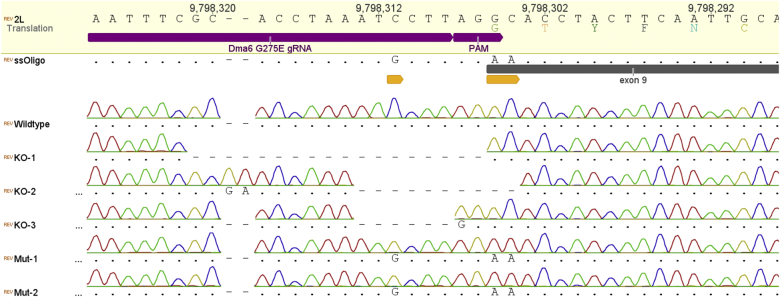
Direct sequencing of Dα6 PCR fragments amplified from gDNA isolated from non-modified flies (wildtype), and spinosad resistant progeny after CRISPR (KO = knock outs, Mut = precise point mutation). The gRNA target site and exon 9 are annotated in purple and grey respectively. Point mutations are highlighted with orange arrows. (For interpretation of the references to colour in this figure legend, the reader is referred to the web version of this article.)

**Fig. 3:**
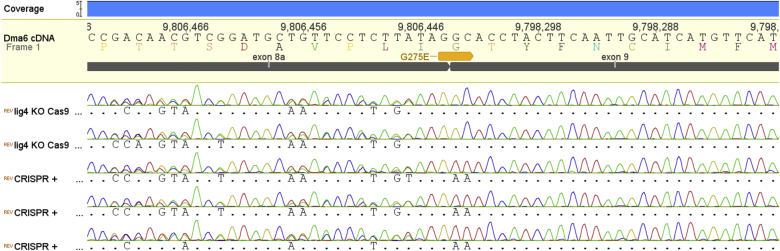
Direct sequencing of Dα6 PCR fragments amplified from cDNA pools isolated from non-modified flies (lig4 KO Cas9), and flies carrying the G275E mutation (CRISPR+) codon position 901–903. Exons 8 and 9 are annotated in grey.

Approximately 250 embryos were injected with a gRNA plasmid and a single stranded oligonucleotide to serve as a template for HDR. The 41 flies that developed from injected embryos (∼16%) were crossed to Dα6 KO flies in vials containing spinosad supplemented media (see Material and Methods) in order to kill progeny carrying the native Dα6 subunit that are highly susceptible to spinosad. 10 of those crossings produced viable offspring (∼25%). 100 Cy males were backcrossed to Cy virgin Dα6 KO females and analysed by PCR and direct sequencing 5 days after the crossing. Sequencing of the genomic loci of interest revealed progeny with a variety of INDELS and others with the desired nucleotide editing. In the case of the former, the various insertions and deletions resulted in gene knock outs either by the introduction of premature stop codons, frame shifts or by loss of splice recognition sites (AG). In the case of the targeted nucleotide replacement all three desired point mutations were observed at the correct positions with the two in exon 9 of the *Dα6* gene conferring the G275E replacement (for representative sequencing traces see Fig. 2). All offspring tested by PCR and sequencing exhibited mutations around the CRISPR/Cas9 target site and the ratio of INDEL mutations mediated by NEJR to HDR mediated insertion of the oligo template was 40:60 (based on 85 viable crossings and PCR reactions). Sequencing of cDNA prepared from CRISPR mutated flies verified the introduced point mutations had not disrupted the normal splicing of the *Dα6* mRNA transcript with the introduced G275E mutation present in transcripts with either exon 8a or 8b, as indicated by the clean peaks for the altered nucleotides in exon 9 and the double peaks present in exon 8 where the two splice forms vary (Fig. 3).

### 3.2 The impact of G275E on the susceptibility of *D. melanogaster* to spinosad in comparison to Dα6 knock out

**Fig. 4:**
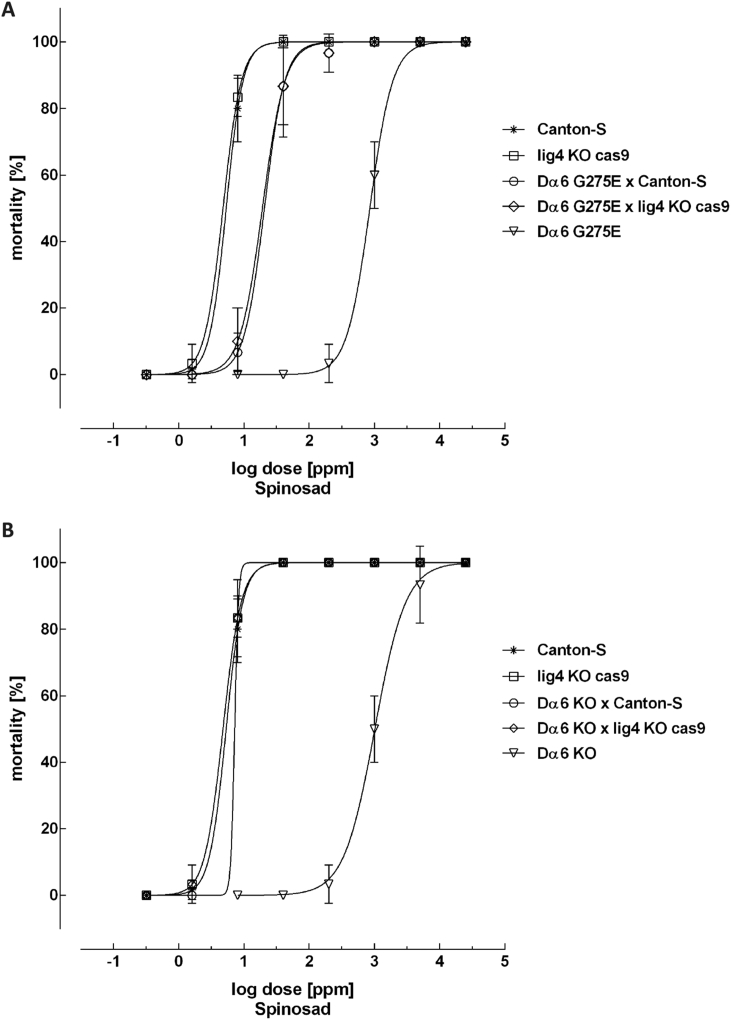
Non-linear log dose-response plots for spinosad against *Drosophila melanogaster* strains and F1 progeny. Error bars represent standard deviation. A) Canton-S, lig4 KO Cas9, Dα6 G275E and F1 progeny of Dα6 G275E × Canton-S and Dα6 G275E × lig4 KO Cas9 respectively. B) Canton-S, lig4 KO Cas9, Dα6 KO and F1 progeny of Dα6 KO × Canton-S and Dα6 KO × lig4 KO Cas9 respectively.

The Dα6 G275E substitution reduces susceptibility significantly when compared to the wild type strain Canton-S and the non-modified strain lig4 KO Cas9 which was the genetic background into which the mutation was introduced. The resistance ratio based on LC_95_ values is ∼66-fold (Table 2 and Fig. 4). Furthermore, two additional independent lines homozygous for the Dα6 G275E substitution were tested and showed a comparable reduced sensitivity to spinosad (Supplementary Table 1). In comparison the Dα6 KO strain deficient for the Dα6 gene exhibited a resistance factor of 311-fold. This is lower than that reported previously by Perry et al. (2007) and likely results from difference in bioassay methodology used in the two studies. Reciprocal crossing with the susceptible strains Canton-S and lig4 KO Cas9 confirmed the completely recessive inheritance of the Dα6 KO (*D* = −1) and revealed an incomplete recessive inheritance of the G275E mutation (*D* = −0.951 based on LC_50_ and *D* = −0.869 based on LC_95_, Table 2).

## 4 Discussion

To date, the G275E mutation has been described in three different insect species (Bao et al., 2014, Puinean et al., 2013, Silva et al., 2016). However, recent studies on *F. occidentalis* strains from China and the USA have failed to detect any nAChR α6 sequence or expression differences between the susceptible and resistant strains prompting the authors to question the role of nAChR α6 subunit in spinosad resistance (Hou et al., 2014). Due to the acknowledged difficulties in expressing insect nAChR in heterologous systems, the role of G275E mutation in spinosad resistance was only inferred from studies done on surrogate receptors (Puinean et al., 2013). In order to establish whether G275E mutation in nAChR α6 subunit plays a direct role in spinosad resistance as suggested in previous studies (Bao et al., 2014, Puinean et al., 2013, Silva et al., 2016), we introduced the homologous mutation in *Drosophila* nAChR α6 receptor using the CRISPR/Cas9 system and compared the phenotypic resistance of mutated versus wild-type/background strain.

**Fig. 5:**
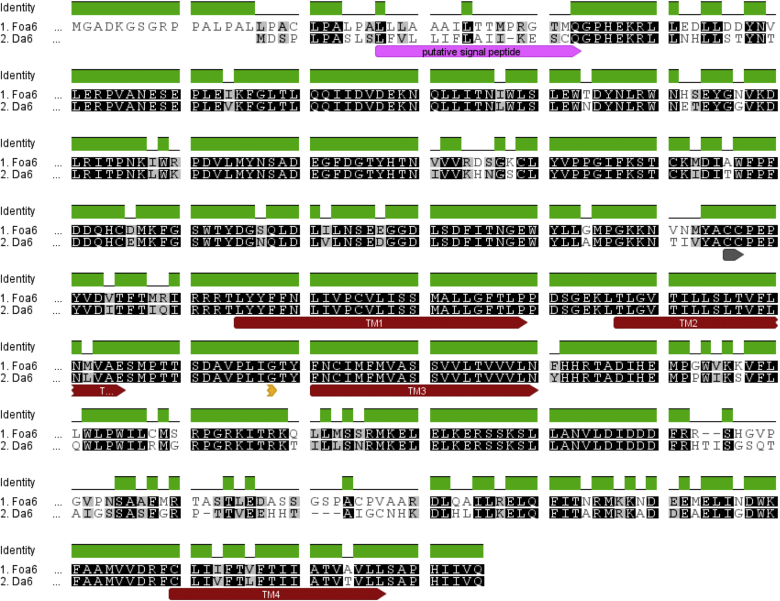
Amino acid sequence alignment of *F. occidentalis* nAChR α6 and *D. melanogaster* nAChR α6 in variant exon 3b/8a. G275E is indicated by an orange arrow and counted from the first amino acid (G) after the putative signal peptide indicated by a pink arrow. Transmembrane regions TM1-4 are annotated with red arrows. (For interpretation of the references to colour in this figure legend, the reader is referred to the web version of this article.)

Genome editing using the CRISPR/Cas9 system has currently only been achieved in a handful of insect species, therefore we used *D. melanogaster* as a surrogate for those species known to carry a target-site mutation conferring spinosad resistance. We believe the use of *Drosophila* as a model in this context is entirely valid as a *Da6* orthologue is typically present in all insect genomes sequenced to date and is highly conserved (Jones and Sattelle, 2010, Jones and Sattelle, 2006, Shao et al., 2007) (Fig. 5). Moreover, it has also been shown that the role of the nAChR α6 in spinosad sensitivity is conserved across species, as α6 orthologues from different species are able to rescue a *Drosophila* spinosad resistant phenotype but not α5 or α7 which are closely related subunits (Perry et al., 2015).

Our results demonstrate that the introduced G275E mutation confers strong resistance in *Drosophila* to spinosad, with the LC_50_ increasing from around 5 mg L^−1^ to 335 mg L^−1^ (Fig. 4). The field rate of spinosad used for control of thrips and tomato leafminer is 87–120 mg L^−1^ and the level of resistance in *Drosophila* provides additional evidence that this mutation alone would be sufficient to result in control failure. Our finding that the G275E mutation is inherited as an incomplete recessive trait in *Drosophila* is in complete concordance with the mode of inheritance of spinosad resistance described in *F. occidentalis* (Bielza et al., 2007) where only homozygous females (or hemizygous males with a single copy of the resistance allele) exhibit sufficient resistance to survive field rates of spinosad. Interestingly, the level of resistance conferred by G275E (66-fold) was lower than for the *Drosophila* line with the *Dα6* null mutation (311-fold). As the nAChR α6 subunit is a redundant insecticide target it is not clear why loss-of-function mutations, which may confer higher resistance, are seen in certain spinosad resistant insect species and specific amino acid substitutions in others. While *Drosophila α6* knock outs are viable and do not display an obvious fitness cost in the lab (Perry et al., 2007), it is likely that there is at least some reduction in fitness associated with loss-of-function mutations in the field environment. In this context a single amino acid substitution would be predicted to carry a lower fitness penalty and may be selected for over more profound genetic alterations in the *α6* gene, if such alterations confer sufficient resistance to survive exposure to recommended field rates. Interestingly, an additional point mutation associated with resistance has very recently been described in *D. melanogaster* where an amino acid replacement, P146S, was identified close to the conserved Cys-loop of the *Dα6* subunit and was shown to confer resistance using the CRISPR/Cas9 system (Somers et al., 2015). In comparison to loss of function mutations, point mutations can often provide new information on the mode and site of insecticide binding at the receptor (Bass et al., 2011, Troczka et al., 2012).

Although the precise location of the spinosad binding site is not known with certainty, homology modelling using the nematode glutamate-gated chloride (Glu-Cl) channel structure predicts the G275E mutation to lie at the top of the third α-helical transmembrane domain of the nAChR a6 subunit (Puinean et al., 2010). A recent crystal structure of the related Cys loop glutamate-gated chloride channel (GluCl) from *Caenorhabditis elegans*, co-crystallized with ivermectin – another macrocyclic lactone insecticide -, reveals that the corresponding amino acid is close (4.4 Å) to the binding site of ivermectin (Hibbs and Gouaux, 2011). In combination with the *in vitro* characterization of the modulatory effect of spinosad on human a7 nAChRs described in the introduction (Puinean et al., 2013) our work provides additional confirmation that the G275E mutation is directly involved in spinosad resistance.

Beyond this study we can see the utility of this model to principally investigate resistance mechanisms in a defined genetic setting. Functional validation of mutations identified in resistant insects is a crucial and yet often missing component of many studies, likely due, in part, to the difficulties involved in expressing insecticide target-sites *in vitro*. In this regard the CRISPR/Cas9 system applied to *Drosophila* is a straight forward and affordable approach to validate putative resistance mutations. Some caution is required as there is no guarantee that introducing SNPs in orthologous genes in *Drosophila* will resemble the exact phenotypes observed in the target organism. Success will likely be dependent on the location of the SNP in the gene of interest and the degree of conservation between orthologous genes of the target organism and the model species.

## Funding

This work was, in part, funded by a research grant (BB/G023352/1) from the Biotechnology and Biological Sciences Research Council of the UK to CB.

## Figures and Tables

**Table 1:** Oligonucleotide sequences for PCR, for cloning into gRNA expression plasmids and template ssOligo for the manipulation of homology directed repair (HDR) in the germline of *D. melanogaster*.

Sequence name	Sequence 5′–3′	5′ overhang
gRNA oligo forward	AATTTCGCACCTAAATCCTT	GTCG
gRNA oligo reverse	AAGGATTTAGGTGCGAAATT	AAAC
G275E ssOligo	TACTGTCAGCACCACCGACGAGGCGACCATGAACATGATGCAATTGAAGTAGGTT TCTAAGCATTTAGGTGCGAAATTTTGGGTTTGCGGATTGCGGATGGCAGTTTGTG	
a6 gDNA PCR F	ATTTTGAGAGACCCCGGAGC	
a6 gDNA/cDNA R	ATATTGTGTGCCGGAAGTCGT	
a6 gDNA seq	ATTGTGTGCCGGAAGTCGTC	
a6 cDNA F1	TGGCACGTATCACACCAACA	
a6 cDNA F2	CATGTACAACAGCGCGGATG	

**Table 2:** Log-dose probit-mortality data for spinosad against *Drosophila melanogaster* strains and F1 progeny.

Strain/Genotype^a^	LC_50_ (mg/L^−1^)	95% CL	LC_95_ (mg/L^−1^)	95% CL	Slope (±SE)	Resistance ratio^b^		Dominance	
LC_50_	LC_95_	LC_50_	LC_95_
Canton-S/+/+	5.7	4.92–7.12	10.04	8.5–12.6	4.254 (±0.279)	1	1		
lig4 KO Cas9/+/+	4.59	4.1–5.1	11.79	10.19–14.16	4.013 (±0.282)	0.8	1.2		
Dα6 G275E/G275E/G275E	354.8	322.93–398.58	665	565.2–827.1	6.029 (±0.499)	62.2	66.2	−0.951	−0.869
Dα6 G275E × Canton-S/G275E/+	22.04	16.39–29.6	85.76	57.08–171.2	2.787 (±0.409)	3.9	8.5	−0.951	−0.869
Dα6 G275E × lig4 KO Cas9/G275E/+	20.73	15.46–27.8	87.68	58.12–172.6	2.626 (±0.364)	3.6	8.7		
Dα6 KO −/−	791	678–923	3122	2453–4293	2.758 (±0.218)	138.8	310.9		
Dα6 KO × Canton-S/−/+	6.17	4.43–8.12	9.6	7.66–12.89	4.431 (±0.289)	1.1	1	−0.999	−1
Dα6 KO × lig4 KO Cas9/−/+	4.21	3.41–5.22	11.21	9.91–14.81	4.023 (±0.341)	0.7	1.1	−1	−1

a nAChR α6 = alleles (diploid) present in this strain, +/+ = homozygous wildtype, −/− = homozygous knock out, G275E/G275E = homozygous mutant.

b Resistance ratio is calculated by dividing LC_50_/LC_95_ of any given strain with the LC_50_/LC_95_ of Canton-S.
