# Supplementary material for: A CRISPR/Cas9 mediated point mutation in the alpha 6 subunit of the nicotinic acetylcholine receptor confers resistance to spinosad in Drosophila melanogaster
Source: Insect Biochem Mol Biol. 2016 Jun;73:62–9. doi: 10.1016/j.ibmb.2016.04.007 (PMC4876769; doi:10.1016/j.ibmb.2016.04.007)
Supplement: Supplementary file 1 [file mmc1.docx]

**Supplementary Fig. 1.** Dose-response relationship in an emergence bioassay with spinosad on lig4 KO Cas9 flies. Spinosad was added to the media at 50 °C, 3 virgin females and males were added to each vial and allowed to propagate for 5 days, emerged flies were scored after 14 days. The bioassay was carried out at 24 °C. None of the doses tested had adulticide effects. Error bars = SD (3 replicates/concentration). A.i. = active ingredient.

**Supplementary Table 1**
Log-dose probit mortality data (48h) for spinosad against three Drosophila melanogaster G275E lines produced via CRISPR/Cas9 gene editing. The three stocks represented in this table were rescued from flies emerging from different rounds of embryo injections and are as such completely independent stocks.

|  | LC_50_ (mg/L^-1^) | 95% CL | LC_95_ (mg/L^-1^) | 95% CL | Slope (± SE) |
| --- | --- | --- | --- | --- | --- |
| G275E Stock 1 | 354.8 | 322.93 - 398.58 | 665 | 565.2 - 827.1 | 6.029 (±0.499) |
| G275E Stock 2 | 387.8 | 340.16 - 445.44 | 906.8 | 750.97 - 1162.69 | 4.458 (±0.244) |
| G275E Stock 3 | 357.3 | 177.66 - 819.91 | 1217.2 | 601.16-14432.49 | 3.090 (±0.161) |
